# Melanocortin-4 Receptor Deficiency Phenotype with an Interstitial 18q Deletion: A Case Report of Severe Childhood Obesity and Tall Stature

**DOI:** 10.1155/2016/6123150

**Published:** 2016-09-21

**Authors:** Sarah Abdullah, William Reginold, Courtney Kiss, Karen J. Harrison, Jennifer J. MacKenzie

**Affiliations:** ^1^School of Medicine, Queen's University, Kingston, ON, Canada; ^2^Department of Medical Imaging, University of Toronto, Toronto, ON, Canada; ^3^Department of Pediatrics, Kingston General Hospital, Kingston, ON, Canada; ^4^Department of Pathology and Laboratory Medicine, IWK Health Centre, Halifax, NS, Canada; ^5^Department of Pathology, Dalhousie University, Halifax, NS, Canada

## Abstract

Childhood obesity is a growing health concern, associated with significant physical and psychological morbidity. Childhood obesity is known to have a strong genetic component, with mutations in the* melanocortin-4 receptor* (*MC4R*) gene being the most common monogenetic cause of obesity. Over 166 different* MC4R* mutations have been identified in persons with hyperphagia, severe childhood obesity, and increased linear growth. However, it is unclear whether the MC4-R deficiency phenotype is due to haploinsufficiency or dominant-negative effects by the mutant receptor. We report the case of a four-and-a-half-year-old boy with an interstitial deletion involving the long arm of chromosome 18 (46,XY,del(18)(q21.32q22.1)) encompassing the* MC4R* gene. This patient presented with tall stature and hyperphagia within his first 18 months of life leading to significant obesity. This case supports haploinsufficiency of MC4-R as it describes a MC4-R deficiency phenotype in a patient heterozygous for a full* MC4R* gene deletion. The intact functional allele with MC4-R haploinsufficiency has the potential to favor a therapeutic response to gastric surgery. Currently, small molecule MC4-R agonists are under development for pharmacologic therapy.

## 1. Introduction

Childhood obesity is a global epidemic with grave physical and psychological effects. Worldwide, 42 million children under age 5 are overweight or obese [1]. Overweight children are at greater risk for Type 2 diabetes, hypertension, stroke, sleep apnea, osteoarthritis, and certain cancers [2]. They also suffer from poor quality of life, low self-esteem, and negative stereotyping [3]. Twin, family, and adoption studies have demonstrated a significant genetic component to obesity, with the heritability of BMI being as high as 80% in children [4, 5]. A single gene mutation can be linked to obesity in 5–7% of obese children, and the most common monogenetic cause of childhood obesity is a mutation in the* melanocortin-4 receptor* (*MC4R*) gene [6, 7].

*MC4R* is a single-exon gene on chromosome 18q21.3. It codes for a G-protein coupled receptor expressed mainly in the brain [8]. Activation of MC4-R is anorexigenic resulting in decreased energy intake and catabolic processes. Over 166 different* MC4R *mutations including frameshift, deletion, nonsense, and nonsynonymous have been identified in obese persons [8, 9]. The phenotype common to* MC4R* mutation carriers is hyperphagia within the first year of life leading to severe childhood obesity, increased lean mass, increased linear growth, and extreme hyperinsulinemia [10].

There are inconsistencies in the literature about whether the phenotype caused by* MC4R* mutations is due to haploinsufficiency or dominant-negative effects by the mutated receptor. In haploinsufficiency, there is a loss of function mutation in one copy of* MC4R* and the remaining normal copy is unable to produce sufficient gene product to maintain normal height and body habitus. Haploinsufficiency of MC4-R is supported by* in vitro* assays of* mc4r* mutants and rodent* mc4r* knockouts.* In vitro* receptor function assays of mutant MC4-R activity were unable to detect dominant-negative effects in the majority of* MC4R* mutations [8]. In addition, mice heterozygous for the* mc4r* deletion exhibit weight gain intermediate to homozygous knockout and wild type mice [11]. However, the human data so far has been inconclusive about haploinsufficiency of MC4-R. A study by Farooqi et al. (2003) supported haploinsufficiency of MC4-R by demonstrating that individuals with heterozygous* MC4R* mutations express a less severe phenotype of hyperphagia, obesity, increased lean mass, increased linear growth, and hyperinsulinemia than individuals with homozygous* MC4R* mutations [12]. However, Cody et al.'s (1999) study of terminal 18q deletions was unable to demonstrate a difference in obesity between patients with and without deletions of* MC4R*, suggesting that MC4-R is haplosufficient and that mutant MC4-R proteins may have dominant-negative effects [13].

To demonstrate a causative effect of haploinsufficiency, patients heterozygous for a full* MC4R* gene deletion should have the same phenotype as patients who are carriers of a loss of function mutation in the gene. We report a four-and-a-half-year-old boy with hyperphagia, morbid obesity, and tall stature identified by cytogenetic analysis to have a unique interstitial deletion of chromosome 18 encompassing the* MC4R* gene. This case report describes a patient with a full deletion of one copy of* MC4R *who has a similar phenotype to* MC4R* mutation carriers, supporting the hypothesis that haploinsufficiency of MC4-R is responsible for this phenotype.

## 2. Case Report

**Figure 1:**
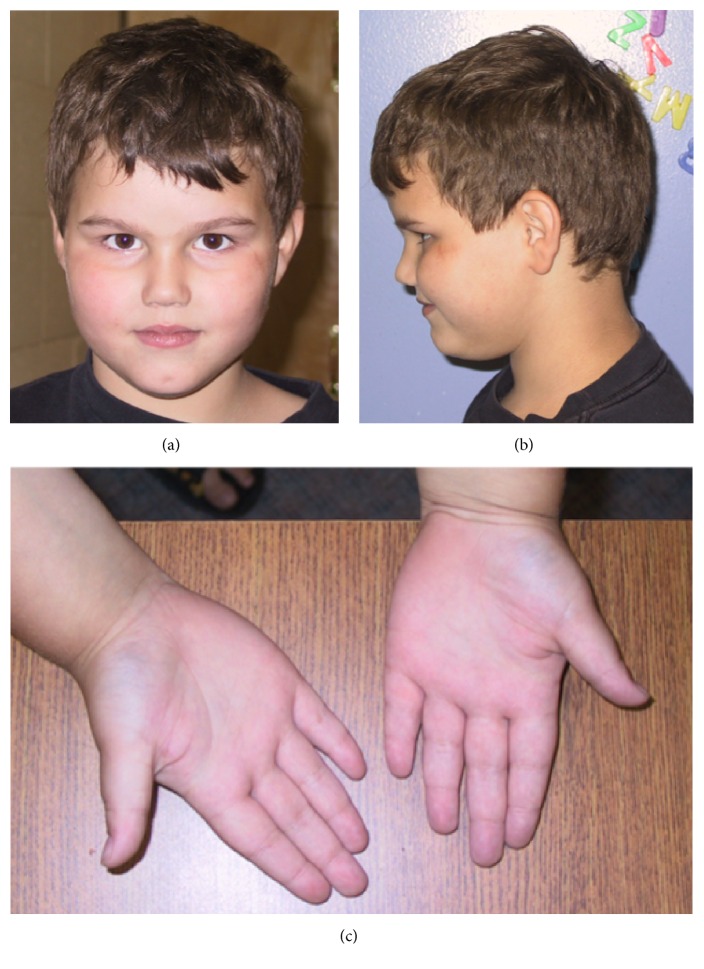
(a) Patient en face. (b) Patient profile. (c) Bridged palmar creases, fifth finger clinodactyly on the left.

A four-and-a-half-year-old boy presented with obesity, tall stature, global developmental delay, and a placid temperament (Figure 1). His birth weight at term was 7 pounds and 4 ounces and growth was poor until 15 months, when he started to gain weight rapidly. By 18 months of age, he had gained a significant amount of weight as he ate frequently and excessively. The patient's parents reported that he did not realize when to stop eating, though he did not forage for food.

**Figure 2:**
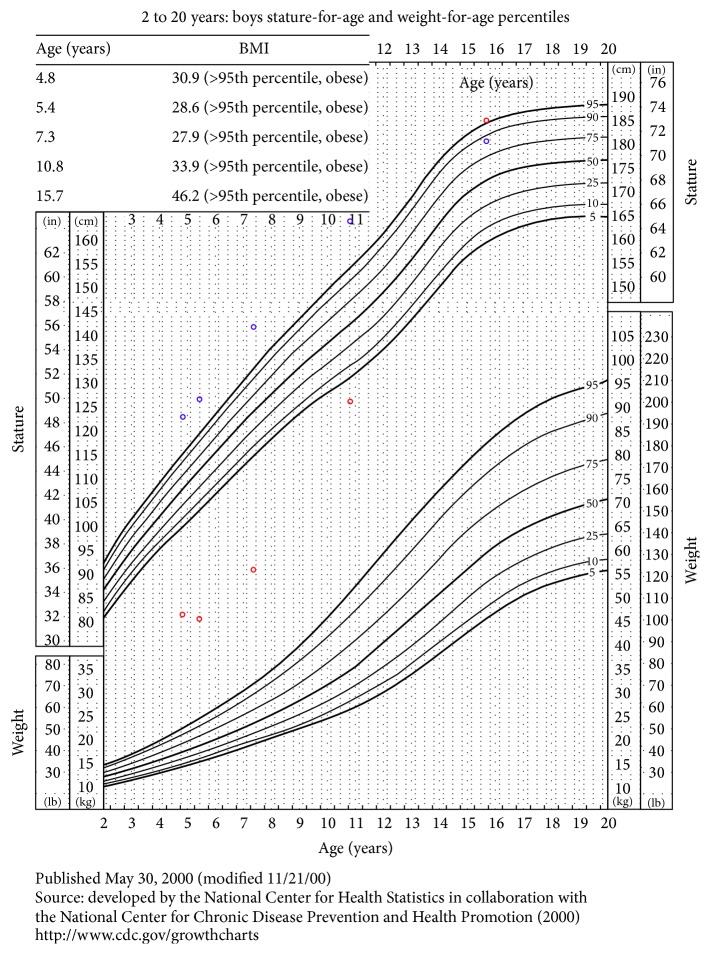
Patient's BMI, stature-for-age growth chart, and weight-for-age growth chart from 4 years to 16 years.

At 4.5 years old, the child's height and weight were greater than 95th percentile, with weight disproportionately larger than height (Figure 2). His head circumference was at 90th percentile. He had a short forehead, well formed ears with fleshy lobes, a prominent brow, and a small dimpled chin. The obesity was associated with striae and inverted nipples. He had bridged palmar creases bilaterally, fifth finger clinodactyly on the left hand, and an increased space between the first and second toes.

At 4.5 years of age, the patient's bone age was 6.5 to 7 years, which is more than 2 standard deviations above the mean for his age. At 10 years of age, he had some pubic and axillary hair. His penis appeared small upon examination at both 10 and 15 years of age. At 15 years of age, the patient's height was 180.7 centimeters (90th percentile), weight was 150.8 kilograms (>97th percentile), and head circumference was 58 centimeters (75th–90th percentile). The BMI was 46.2, which is well above 99th percentile. Hormone studies were not available.

There was a family history of obesity, but not tall stature. The patient's father's BMI was 43.4 (obese), and his mother's BMI was 41 (obese). The patient's older brother's BMI was 26.2 (overweight). The patient's brother reportedly met his early developmental milestones appropriately. Subsequently, the brother had difficulties with speech, reading, and memory processing but was able to progress through school with assistance.

Genetic investigations including methylation studies for Prader Willi syndrome and Southern Blot for FMR1 for the patient were normal. Karyotype results were 46,XY,del(18)(q21.32q21.32). Sequencing of the* MCR4 *gene did not detect any mutations. A CGH microarray was undertaken to further define the patient's deletion.

Microarray evaluation using a 180k CGH microarray platform (hg19) gave a result of arr[hg19] 1p13.3 (109,336,197–109,779,618) × 3; 18q21.32q22.1 (56,201,352–62,696,664) × 1. The 0.463 Mb gain at 1p13.3 was described as a variant of uncertain significance and included the following genes:* STBP3, AKNAD1, GPSM2, CLCC1, WDR47, TAF13, TMEM167B, SCARNA2, C1orf194, KIAA1324, SARS, *and* CELSR2*. The 6.46 Mb loss at 18q is unique in size and location and includes the following genes:* SERPINB3,* ***MC4R*** *, PMAIP1, SERPINB5, VPS4B, SERPINB2,* ***MALT1***, ***BCL2***, ***LMAN1*** *, SCCHC2, PHLPP1, KDSR, SERPINB13, SERPINB10, HMSD, SEPRINB8,* ***SERPINB7*** *, SERPINB11,* ***TNFRSF11A*** *, SERPINB4, SERPINB12, KIAA1468,* ***RAX*** *, CPLX4, ALPK2, SEC11C, ZNF532,* ***CCB3E1*** *, GRP, CDH20, RNF152, *and ***PIGN***. Of the preceding list, the OMIM morbid disease genes are in bold. Deletion of* MALT1, LMAN1, SERPINB7, TNFRSF11A, RAX, CCB3E1, *and* PIGN *was not expected to have an effect on the patient as loss of function mutations need to be present in both copies of these genes in order to produce a disease state [14]. Heterozygous mutations in Bcl-2 in mice have been shown to produce manic behavior, although this was not seen in our patient [15]. Parental testing revealed that the 18q deletion was* de novo*. The patient's mother also had the 1p13.3 duplication.

Physical activity and diet management were recommended to manage the patient's obesity. He participated in a gym class and was counseled to minimize his excessive intake of sugar and unhealthy foods. In addition, the patient's family doctor was advised to monitor the patient for complications of obesity, including hyperinsulinemia, diabetes, and heart disease.

## 3. Discussion

This case links the 18q deletion to a MC4-R deficiency phenotype of obesity. Cody et al. (1999) were previously unable to demonstrate a difference in weight or height between patients with terminal 18q deletions including or sparing* MC4R* [13]. It is possible that the severe childhood obesity and tall stature in our patient may be coincidental with his 18q deletion. Alternatively, our patient may have manifestation of the MC4-R deficiency phenotype because his deletion is interstitial compared to terminal deletions containing* MC4R*, which are typically characterized by short stature [16]. A number of genes are present downstream of* MC4R* on chromosome 18 and may potentially modify the phenotype in patients with terminal deletions. For example, terminal 18q critical regions for short stature and growth hormone insufficiency have been identified in 18q22.3-q23 [16]. Our patient's deletion did not include these regions. Feenstra et al. (2007) report that patients with deletions distal to 18q21.33 tend to have mild intellectual disability (consistent with our patient's deletion and phenotype), while patients with deletions proximal to 18q21.31 tend to have more severe intellectual disability. Future studies using high-resolution techniques have the potential to confirm the MC4-R deficiency phenotype in patients with interstitial 18q deletions encompassing* MC4R* and to characterize which genes downstream of* MC4R *modify the obesity phenotype.

Since there is only one copy of* MC4R* in our patient, this case supports haploinsufficiency as the cause of the obesity phenotype. A model of MC4-R haploinsufficiency is in agreement with rodent* mc4r* models as well as human data collected by Farooqi et al. (2003) [11, 12]. A review of deletions at 18q21.32q22.1 in the Decipher database identified 3 patients with deletions encompassing* MC4R*. Decipher patient 248187 presented with truncal obesity and intellectual disability, similar to our patient. Decipher patient 249747's clinical features included facial asymmetry and spasticity, which were not seen in our case. Decipher patient 264313 presented with intellectual disability. The latter 2 Decipher patients did not exhibit the obesity phenotype associated with* MC4R* mutations. This may be because the other genes present in their deletions may have modified their obesity phenotype. Alternatively, these patients may not have been obese because heterozygous* MC4R *mutations have a penetrance of only 63.5% [17].

Recently, Turner et al. (2015) described a patient with a 2.9 Mb deletion at [hg18] 18q21.31 [18]. This patient's deletion encompassed* MC4R* and he presented with obesity. This patient's father also had the 18q21.31 deletion, but was not obese. This case report suggested a possible link between the* MC4R* deletion and attention deficit hyperactivity disorder (ADHD) because a number of individuals on the patient's father's side were reported to have ADHD [18]. However, our patient does not have any signs of ADHD, nor a family history of ADHD.

The importance of our case is that it shows the obesity phenotype seen in* MC4R* mutation carriers in a patient heterozygous for a full* MC4R* gene deletion. This indicates that MC4-R is haploinsufficient and that it is unlikely that mutant MC4-R proteins exert dominant-negative effects. This hypothesis is consistent with* in vitro* assays of mutant MC4-R activity that were unable to detect dominant-negative effects in most* MC4R* mutations [8].

Haploinsufficiency of MC4-R should be more amenable to treatment than dominant-negative effects. In the rodent* mc4r* model, voluntary running wheel activity slowed down the course of obesity [19]. Therefore, as was done for the patient in this case report, all patients with* MC4R* mutations should be advised that lifestyle modification can oppose the obesity effect from MC4-R deficiency. Roux-en-Y gastric bypass is more effective at producing weight loss in* mc4r* model mice that have a single normal copy of* MC4R* [20]. Since MC4-R is haploinsufficient, patients carrying a single mutation in* MC4R* should have the same response to gastric bypass as patients without mutations [21]. Haploinsufficiency can be also successfully treated with agonists for the remaining normal functioning allele. Several promising MC4-R agonists are under development and will be entering phase I and phase II clinical trials [22, 23].

## 4. Conclusion

It has previously been unclear whether mutations in the* MC4R* gene produce a phenotype of childhood obesity by haploinsufficiency or dominant-negative effects of a mutant MC4-R protein. This case report supports a mechanism of haploinsufficiency by demonstrating that individuals with a deletion of a single copy of* MC4R* can present with hyperphagia, severe childhood obesity, and tall stature. Haploinsufficiency of MC4-R has the potential to be treated with current surgical treatments and future pharmacologic therapies.

## Abbreviations

MC4-R: Melanocortin 4 receptor protein M
*MC4R*: Melanocortin 4 receptor gene
BMI: Body Mass Index
CGH: Comparative Genomic Hybridization
FMR1: Fragile X mental retardation 1
Mb: Megabases (of DNA)
ADHD: Attention deficit hyperactivity disorder.
